# MicroRNA MiR-130a-3p promotes gastric cancer by targeting Glucosaminyl N-acetyl transferase 4 (GCNT4) to regulate the TGF-β1/SMAD3 pathway

**DOI:** 10.1080/21655979.2021.1995099

**Published:** 2021-12-07

**Authors:** Wei Hu, Xin Zheng, Jun Liu, Min Zhang, Yan Liang, Ming Song

**Affiliations:** Department of General Surgery, Wuhan Third Hospital, Wuhan, Hubei, China

**Keywords:** Mir-130a-3p, gcnt4, gastric cancer, proliferation, migration

## Abstract

Gastric cancer is the third-leading cause of cancer-related deaths worldwide. Dysregulation of glucosaminyl (N-acetyl) transferase 4 (GCNT4) gene and miR-130a-3p gene has been reported in the development of gastric cancer. We elucidated the function of the miR-130a-3p-GCNT4 axis in gastric cancer. Reverse transcription quantitative polymerase-chain reaction measured miR-130a-3p and GCNT4 levels in gastric cancer tissues and cells. The interaction between miR-130a-3p and GCNT4 was assessed using luciferase and RNA pull-down assays. Biological roles of miR-130a-3p and GCNT4 were determined using cell proliferation, migration, and invasion assays in gastric cancer cells. In addition, the effect of miR-130a-3p on the tumor growth in vivo was investigated using tumor xenografts assay. Levels of total TGF-β1, phosphorylated SMAD3 (p-SMAD3), and SMAD3 were measured by using western blot. The results showed that miR-130a-3p levels were increased, while GCNT4 levels were reduced in gastric cancer tissues and cell lines. While miR-130a-3p mimics facilitated cellular proliferation, migration, and invasion in vitro, promoted tumor growth in vivo, and activated the TGF-β1/SMAD3 signaling pathway, overexpression of GCNT4 prevented the growth of gastric cancer cells and restrained the activation of the TGF-β1/SMAD3 pathway. Mechanistically, miR-130a-3p suppressed gastric cancer genesis by inhibiting GCNT4 expression and activating the TGF-β1/SMAD3 signaling pathway. Altogether, we proposed that targeting of GCNT4 and activation of the TGF-β1/SMAD3 signaling pathway by miR-130a-3p enhanced the growth of gastric cancer cells. This study provides important strategies for the selection of therapeutic targets for gastric cancer treatment involving miR-130a-3p/GCNT4/TGF-β1/SMAD3 axis.

## Background

Gastric cancer is a highly common malignancy, which is the third-leading cause of cancer-related deaths worldwide [1], and as reported by from the latest statistics, it causes serious mortality in China [2]. Although therapeutic strategies for gastric cancer have benefited from surgery, chemotherapy, and radiotherapy in the recent years, the 5-year survival rate remains unsatisfactory [3]. Therefore, identifying effective biomarkers for gastric cancer treatment is essential.

MicroRNAs (miRNAs) play important biological roles in cellular growth and apoptosis by targeting the 3ʹ-untranslated region (3ʹ-UTR) of genes [4]. miRNAs promote tumorigenesis or tumor suppression depending on the function of the target genes [5,6]. miR-130a-3p affects tumor progression in various cancers, including cervical cancer, renal cell carcinoma, and lung cancer [7–9]. Two studies have suggested that miR-130a-3p facilitates tumorigenesis by regulating phosphatase and tensin homolog and p21 expression while acting as a suppressor by inhibiting TBL1X receptor 1 expression in human gastric cancer [10,11]. However, functions of both miR-130a-3p and GCNT4 in the development of gastric cancer remain unknown.

The glucosaminyl (N-acetyl) transferase 4 (GCNT4) gene is located on chromosome 5q13.3 and consists of four exons – GCNT1, GCNT2, GCNT3, and GCNT4, of the GCNT family, which are important in regulating synthesis, branching, and oligomerization of the mucin core structure [12]. It influences cancer genesis by regulating cell growth and apoptosis in pancreatic, prostate, and colon cancer [13–15]. One study suggested that GCNT4 was significantly downregulated in gastric cancer and was associated with a poor prognosis. In a functional study, overexpression of GCNT4 could repress cell proliferation and the cell cycle [16]. However, the mechanism of the interaction between miR-130a-3p and GCNT4 in gastric cancer requires further evaluation.

TGF-β is a class of cytokines with multiple biological functions [17], which participates in a variety of complex physiological and pathological processes, including cancer by influencing cell proliferation, migration, and invasion [18]. SMAD3 is an important TGF-β signaling molecule, which can be phosphorylated by high expression of TGF-β1, thus affecting cell behavior [19]. TGF-β1 inhibitors have been reported to block migration, invasion, and epithelial–mesenchymal transformation of gastric cancer cells and inhibit the phosphorylation of SMAD3 [20]. Therefore, it is particularly important to study changes in the TGF-β1/SMAD3 signaling pathway in gastric cancer.

Thus, in this study, we investigated the effects of miR-130a-3p and GCNT4 in human gastric cancer cell lines and normal gastric epithelial GES-1 cells. We hypothesized that miR-130a-3p facilitates gastric cancer progression by downregulating GCNT4, which blocks the activation of the TGF-β1/SMAD3 signaling pathway. The findings of this study have important implications for the selection of therapeutic targets for gastric cancer treatment involving miR-130a-3p/GCNT4 and TGF-β1/SMAD3 axes.

## Methods

### Bioinformatics analysis

GSE116312 from GEO DataSets (www.ncbi.nlm.nih.gov/gds) contains mRNA expression data from gastric cancer and non-tumor samples. With the selection criteria of adjusted P < 0.05, and logFC ≤ −1.5, differentially expressed genes (DEGs) in gastric cancer samples were filtered out. Then, the STRING algorithm (string-db.org) performed a protein–protein interaction analysis for the DEGs. GSE93415, also from GEO DataSets, contains miRNA expression data from gastric cancer samples and non-tumor samples. With the selection criteria of adjusted P < 0.05 and logFC ≥ −1.5, the upregulated miRNAs in gastric cancer samples were filtered out. At the same time, TargetScan (www.targetscan.org/vert_71) was used to predict the miRNAs targeting the key gene GCNT4. Finally, the common miRNAs in GSE93415 and TargetScan were identified using Venny 2.1.0 (bioinfogp.cnb.csic.es/tools/venny).

### Human samples collection, cells culture, and transfection

A total of 45 gastric cancer patients from our hospital contributed their tissues and adjacent normal tissues with informed consent, which was approved by the ethics committee of Wuhan Third Hospital. Human gastric cancer cell lines (HGC-27, MKN45, and AGS) and normal gastric epithelial GES-1 cells were obtained from the American Type Culture Collection (USA). All the cells were cultured in the 10% fetal bovine serum (FBS)-containing RPMI-1640 medium (Gibco, USA) at 37°C and 5% CO_2_ condition. MiR-130a-3p mimic, mimic-negative controls (NC), miR-130a-3p inhibitor, inhibitor-NC, overexpression-GCNT4, and empty vector were purchased from Tuoran Bio (Shanghai, China). After cells reached up to 30% density, the MKN45 and AGS cells were transfected using Lipofectamine 3000 (Thermo, USA) for 48 h.

### RNA extraction and RT-qPCR analysis

Total mRNA from gastric cancer tissues and cells was isolated using TRIzol reagent (Thermo, USA), cDNA was synthesized using transScript One-Step gDNA Removal and cDNA Synthesis SuperMix (Cat#: AT311-02, transgene, China), and RT-qPCR was performed using SYBR Premix Ex Taq (Cat#: #RR420A Takara, China). miRNA was isolated using the miRNeasy mini kit (Cat#: #217,004, QIAGEN, Germany), cDNA was synthesized using miScript II RT Kit (Cat#: #218,161, QIAGEN, USA), and RT-qPCR was performed using miScript SYBR Green PCR Kit (Cat#: #218,075, QIAGEN, USA). GCNT4 expression relative to glyceraldehyde-3-phosphate dehydrogenase (GAPDH) and miR-130a-3p levels relative to Uracil6 (U6) were estimated by the 2-^ΔΔCt^ method [21]. Primer sequences are listed in Table 1.

**Table 1.** The sequences of the PCR primers in this study

Primer	Sequences
**miR-233-3p**	Forward: 5ʹ-CGCUAUCUUUCUAUUAACUGACCAUAA-3ʹ
	Reverse: 5ʹ-CGCUAUCUUUCUAUUAUGACUCCAUAA-3ʹ
**miR-135b-5p**	Forward: 5ʹ-CGGGCTATGGCTTTTTATTCC-3ʹ
	Reverse: 5ʹ-CAGCCACAAAAGAGCACAAT-3ʹ
**miR-130a-3p**	Forward: 5ʹ-TCGTGGGCAGTGCAATGTTAAAA-3ʹ
	Reverse: 5ʹ-CAGTGCTGGGTCCGAGTGA-3ʹ
**GCNT4**	Forward: 5ʹ-AGGCTCCTCTTCCCTCAAAG-3’
	Reverse: 5ʹ-ACATCACCGTCCTCCAAGTC-3’
**GAPDH**	Forward: 5ʹ-TCCTGCACCACCAACTGCTT-3’
	Reverse: 5ʹ-AGGGGCCATCCACAGTCTTC-3’
**U6**	Forward: 5ʹ-CTCGCTTCGGCAGCACA-3’
	Reverse: 5ʹ-AACGCTTCCACGAATTTGCGT-3’

### Cell proliferation assay using Cell Counting kit 8 (CCK-8)

Cell viability was examined using the CCK-8 kit (Cat#: C0037; Beyotime, China). MKN45 and AGS cells (5 × 10^3^) were cultured in transparent 96-well plates. Cells were counted at 0, 24, 48, and 72 h. Two hours before every time point, 10 µL CCK-8 working buffer was added to the wells and incubated at 37°C. Finally, each well was evaluated by measuring the OD at 450 nm on a multimode-plate reader (Thermo, USA) [22].

### Cell proliferation using 5ʹ-Bromo-2ʹ-deoxyuridine (BrdU) assay

Cell proliferation was determined using a BrdU kit (Cat#: 6813, CST, USA). Transfected MKN45 and AGS cells (5 × 10^3^) were cultured in transparent 96-well plates. At 80% cell density, cells were washed, denatured, and incubated with BrdU antibody for 2 h at 25°C. Then, the secondary antibody was added to each well and incubated for 1 h at 25°C. Finally, each well was evaluated by measuring the OD at 450 nm on a multimode-plate reader (Thermo, USA) [23].

### Cell migration assay

MKN45 and AGS cells (1 × 10^6^) were cultured in 6-well plates. The cell culture medium was changed to 2% FBS-containing medium at 50% density, and a 10 μL sterile pipette was used to make the artificial scratch and washed twice. Images of wound recovery were photographed under a light microscope at 0 and 24 h. The diameter of cells was measured and recorded [9].

### Transwell assay

Transfected MKN45 and AGS cells (95 × 10^4^ cells) were seeded in the upper chamber (Cat#: #3244, Coring, USA) without serum and precoated with Matrigel (2 μg/well, BD, USA). A complete cell culture medium containing 10% FBS was added to the lower chamber. After 48 h, cells were removed from the upper chamber, and cells in the lower chamber were fixed using 4% paraformaldehyde for 10 min, followed by the treatment with 0.1% crystal violet for 10 min at 25°C. Finally, stained cells were photographed and counted under a microscope (Olympus, Tokyo, Japan) [9].

### In vivo *xenograft model*

This study was carried out in accordance with guidelines for the care and use of laboratory animals. The animal experiment program was approved by the research ethics committee of our hospital. 5 × 10^5^ MKN45 cells were suspended in 100 µL of medium and injected subcutaneously into BALB/c nude mice purchased from the Guangdong Experimental Animal Center (Guangdong, China). Next, 100 nmol/kg miR-130a-3p antagomiR (antagomiRNA) and antagomiR-NC (negative control) were administered through the tail vein every 48 h. On the 7th day after the injection, the tumor size was measured with calipers on each day of the week as follows: volume = width^2^ × length × 0.5. On the 29th day of cell inoculation, the mice were euthanized, and the tumor was removed and weighed [24].

### Luciferase assay

The pmiRGLO vectors containing wild-type (WT) or mutant (MUT) GCNT4 3ʹ-UTRs sequences were purchased from Genechem (Shanghai, China). The MKN45 and AGS cells were treated with 0.24 µg of either vector and 40 nM ofmiR-130a-3p or miR-130a-3p NC using Lipofectamine 3000. After 72 h of transfection, the Luciferase Assay Kit (Cat#: RG027; Beyotime, China) was used for dual-luciferase activity measurements. The final results were represented by firefly luciferase activities relative to that of Renilla luciferase [22,25].

### RNA-pull down analysis

Biotin-labeled MiR-130a-3p (Bio-miR-130a-3p) and miR-negative control (Bio-NC) were designed and synthesized by Thermo Fisher (USA). MKN45 and AGS cells were lysed and subsequently incubated with Bio-miR-130a-3p or Bio-NC for 2 h. Next, the suspension was incubated overnight with streptavidin beads (Sigma, USA) at 4°C. Beads were washed and the elution was purified with an RNA purification Kit (Cat#: DP412, Tiangen, China). After the enrichment, GCNT4 was quantified by RT-qPCR [26].

### Western blotting analysis

Transfected MKN45 and AGS cells were lysed in radioimmunoprecipitation assay (RIPA) buffer (Cat#: P0013B, Beyotime, China) and subsequently denatured at 95°C for 5 min. Equal amounts of protein from each sample were separated on 10% SDS-PAGE at 80 V for 2 h. Then, proteins were transferred to a polyvinylidene fluoride membrane at 72 V for 1.5 h. After blocking in Tris-buffered saline with Tween 20 containing 5% bovine serum albumin, membranes were incubated with primary antibodies against GCNT4 (1:1000, Cat#: PA5-57,950, Thermo Fisher, USA), TGF-β1 (1:1,000, Cat#: ab92486), Smad3 (1:1,000, Cat#: ab40854), phospho-Smad3 (1:1,000, Cat#: ab52903), and GAPDH (1:2000, Cat#: SAB1410512, Sigma, USA) overnight at 4°C. After washing thrice, membranes were incubated with the anti-HRP-Rabbit antibody for 1 h at 25°C. The results were detected by a chemiluminescence (ECL) system (#32,209, Thermo Fisher, USA). GCNT4 protein levels were normalized to those of the GAPDH [9].

## Statistical analysis

Statistical analysis was carried out based on three independent experiments using GraphPad software (version 8.0; GraphPad, USA). Paired Student’s *t*-test was used for two-group analyses, and a one-way analysis of variance test was used for multiple group analyses. Data are presented as mean ± standard deviation (SD). Pearson correlation analysis was used to study the expression of GCNT4 and miR-130a-3p in gastric cancer tissues. *P* < 0.05 was considered statistically significant.

## Results

Using bioinformatics analysis, miR-130a-3p and GCNT4 were identified as the genes of interest with respect to gastric cancer. We aimed to provide important insights for the treatment of gastric cancer. Therefore, we investigated the expression of miR-130a-3p and GCNT4 in gastric cancer tissues and their effects on the proliferation, migration, and invasion of the cancer cells. We hypothesized that miR-130a-3p promotes the progression of gastric cancer by downregulating GCNT4, which blocks the activation of the TGF-β1/SMAD3 signaling pathway.

### GCNT4 can be potentially suppressed by miR-130a-3p thus promoting gastric cancer progression

**Figure 1.**
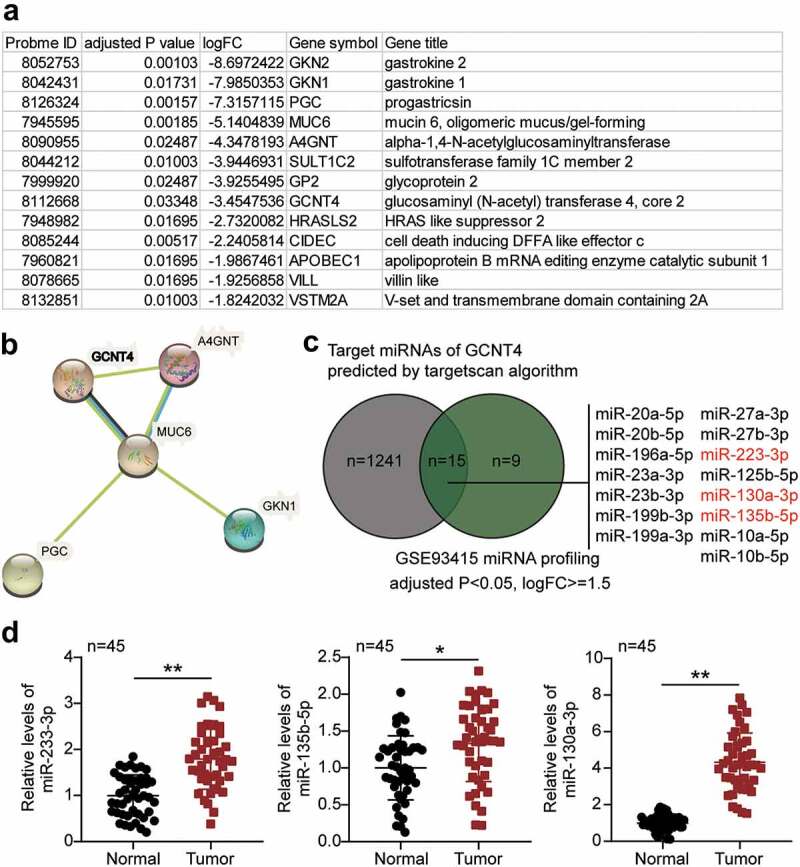
The effects of miR-130a-3p and GCNT4 in gastric cancer were to be studied

Based on the analysis of the GSE116312 data series, 13 DEGs were identified based on the selection criteria of adjusted P < 0.05, and logFC ≤- 1.5 (Figure 1a). The 13 genes were subjected to protein–protein interaction analysis using the STRING algorithm. Results showed that GCNT4, A4GNT, MUC6, PGC, and GKN1 were closely associated (Figure 1b). Among the five interacting genes, GCNT4 has been extensively studied in human cancer, including gastric cancer, and has been reported to suppress gastric cancer cell proliferation phenotype only once [16]. On the contrary, other phenotypes have not been studied. To identify a potential upstream miRNA regulator of GCNT4 in gastric cancer, we selected a list of TargetScan-predicted targets of GCNT4 from the GSE93415 data series, an miRNA microarray profile in gastric cancer. Fifteen miRNAs were shortlisted (Figure 1c), and among them, miR-233-3p, miR-135b-5p, and miR-130-3p were found to be significant tumor promoters in gastric cancer. To verify the miRNAs of interest, we checked the expression of miR-233-3p, miR-135b-5p, and miR-130a-3p in gastric cancer tissues. Results demonstrated that miR-130a-3p was the most significantly upregulated (Figure 1d). Moreover, miR-233-3p and miR-135b-5p have been extensively studied in gastric cancer, whereas miR-130-3p has been relatively unexplored in GC. Thus, we speculated that miR-130a-3p might promote GC progression by targeting GCNT4.

(A) The differentially expressed genes list in gastric cancer by analyzing GSE116312 data series. Selection criteria: adjusted P < 0.05, and logFC≤-1.5. Thirteen genes were identified. (B) The protein–protein interaction network of the 13 genes is analyzed by STRING algorithm. (C) The intersection between the predicted target miRNAs of GCNT4 by Targetscan and the differentially expressed miRNAs analysis of GSE93415 data series (adjusted P < 0.05, logFC ≥ 1.5). (D) RT-qPCR detection of miR-233-3p, miR-135b-5p, and miR-130-3p expression in gastric cancer tissues (n = 45), and normal tissues (n = 45).

### miR-130a-3p promoted gastric cancer genesis

**Figure 2.**
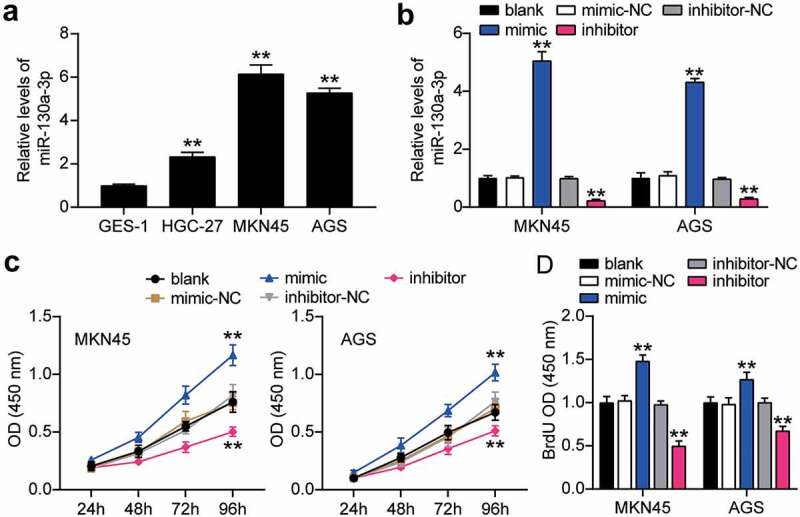
MiR-130a-3p promoted cell viability and proliferation of gastric cancer cells. (A) Measurement of miR-130a-3p expression in gastric cancer cell lines (HGC-27, MKN45 and AGS) and normal gastric epithelial GES-1 cells. (B) Measurement of miR-130a-3p expression in MKN45 and AGS cells transfected with NC and miR-130a-3p mimic by RT-qPCR. (C) Cell viability was detected in MKN45 and AGS cells transfected with miR-130a-3p mimic and NC by CCK-8 assay. (D) Cell proliferation was detected in MKN45 and AGS cells transfected with miR-130a-3p mimic and NC by BrdU assay. *, *P* < 0.05; **, *P* < 0.001. NC, negative control; mimic, miR-130a-3p mimic

**Figure 3.**
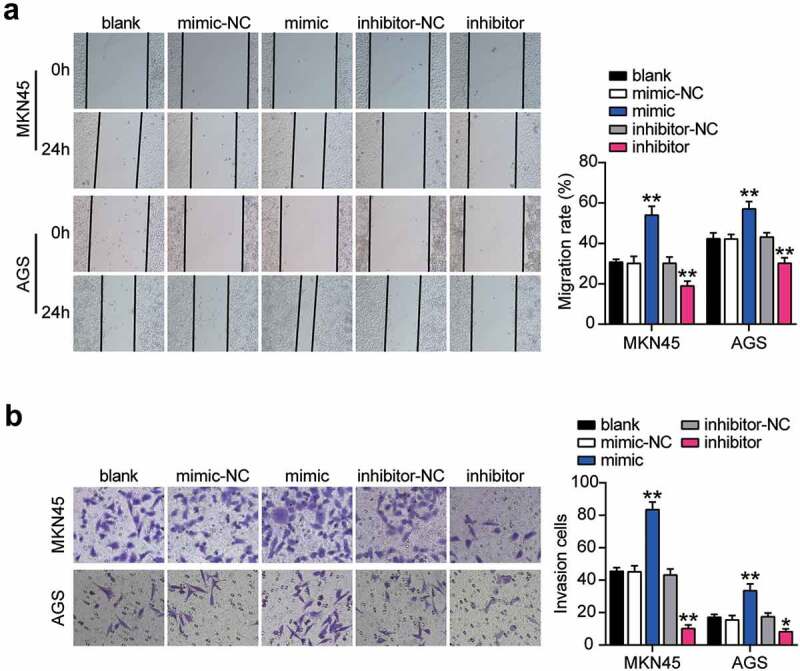
MiR-130a-3p enhanced cell migration and invasion of gastric cancer cells. (A) Cell migration level was determined in MKN45 and AGS cells transfected with NC and miR-130a-3p mimic by wound healing assay. (B) Cell invasion was detected in MKN45 and AGS cells transfected with miR-130a-3p mimic and NC by transwell assay. *, *P* < 0.05; **, *P* < 0.001. NC, negative control; mimic, miR-130a-3p mimic

To verify the abnormal regulation of miR-130a-3p in gastric cancer, we measured miR-130a-3p expression in gastric cancer cells. miR-130a-3p expression was increased in gastric cancer cell lines (HGC-27, MKN45, and AGS) than that in normal gastric epithelial GES-1 cells, especially in MKN45 and AGS cells, which were chosen for subsequent studies (Figure 2a). We further transfected miR-130a-3p mimic or inhibitor into MKN45 and AGS cells. Mimic groups revealed nearly 4-fold miR-130a-3p expression compared with that in mimic-NC cells, while inhibitor groups revealed a downregulation of miR-130a-3p expression compared with inhibitor-NC cells in both MKN45 and AGS cells (Figure 2b). Cell viability was dramatically upregulated in the mimic groups and downregulated in the inhibitor groups in both cell lines (Figure 2c). Simultaneously, mimic groups displayed approximately 1.5-fold enhanced cell proliferation, while the inhibitor groups showed decreased proliferation in both cell types (Figure 2d). Additionally, mimic groups presented an approximately 2-fold and 1.3-fold increase in cell migration, while inhibitor groups presented approximately 50% and 60% migration compared to that in the corresponding control MKN45 and AGS cells, respectively (Figure 3a). Moreover, mimic groups displayed approximately 2-fold upregulated cell invasion, while the inhibitor groups demonstrated decreased cell invasion than that in the control cells (Figure 3b). Taken together, these data demonstrate that miR-130a-3p facilitates cell proliferation, migration, and invasion in gastric cancer cells.

### *miR-130a-3p interference suppressed gastric cancer cell tumorigenicity* in vivo

**Figure 4.**
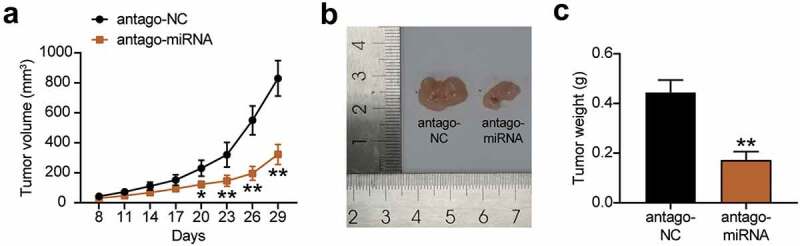
Interference with miR-130a-3p suppressed gastric cancer cell tumorigenicity in vivo. (a) Growth curves for tumor volumes in xenografts of nude mice in antagomiR and antagomiR-NC group. (b) Representative images of tumors in the antagomiR and antagomiR-NC groups. (c) Tumor weights of tumor tissue in antagomiR and antagomiR-NC group. *, *P* < 0.05; **, *P* < 0.001. NC, negative control; antago-miRNA, antago miR-130a-3p

We further studied the tumorigenic effect of miR-130a-3p knockdown on gastric cancer cells in vivo. The results communicated that after the injection of miR-130a-3p antagomiR-treated MKN45 cells into nude mice, the growth in the tumor volume was slower than that of the antagomiR-NC treated cells (Figure 4a). In addition, tumor size and weight were significantly reduced in the antagomiR group than in the antagomiR-NC group (Figure 4b and c). These results suggest that the knockdown of miR-130a-3p reduces the tumorigenicity of gastric cancer cells in vivo.

### GCNT4 is a target of miR-130a-3p

**Figure 5.**
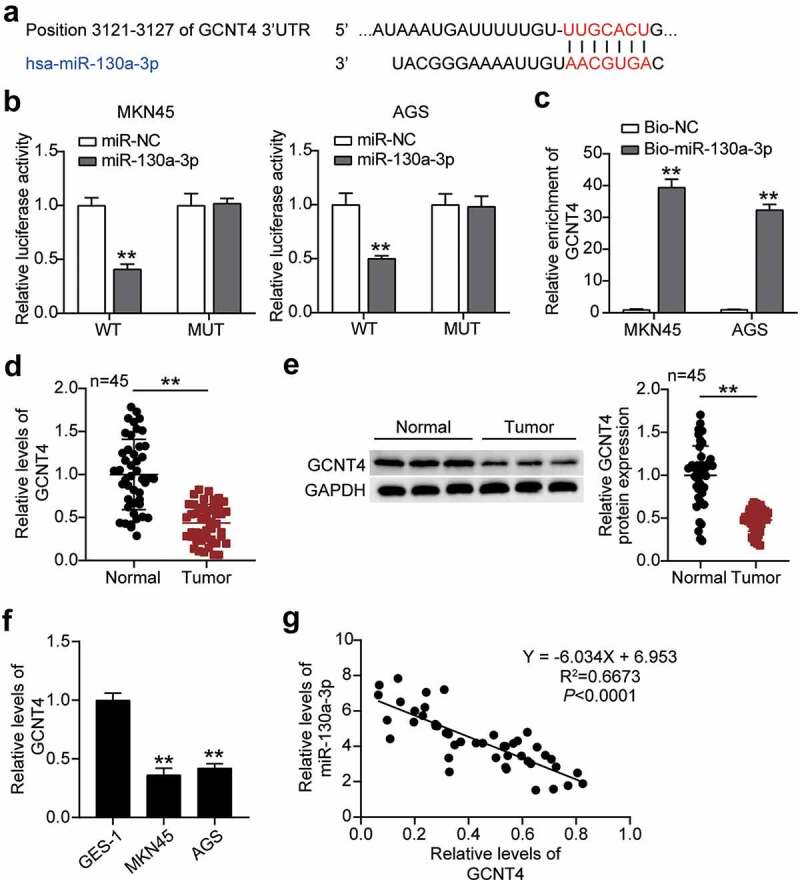
MiR-130a-3p targeting GCNT4 and inhibited the expression of GCNT4. (A) Bioinformatics analysis of the binding site sequence of miR-130a-3p and GCNT4 3ʹ-UTR. (B) Dual-luciferase assay was performed in cells co-transfected with plasmids GCNT4-WT or GCNT4-MUT and miR-NC or miR-130a-3p mimic in MKN45 and AGS cells. (C) RT-qPCR detection of expression of GCNT4 in MKN45 and AGS cells transfected with Bio- miR-130a-3p NC or Bio- miR-130a-3p mimic. (D) RT-qPCR detection of expression of GCNT4 in the gastric cancer tissues and normal tissues. (E) Wester blot detection of protein expression of GCNT4 in the gastric cancer tissues and normal tissues. (F) Measurement of GCNT4 expression in gastric cancer cells lines MKN45 and AGS and normal gastric epithelial GES-1 cells. (G) Correlation analysis between the miR-130a-3p expression and GCNT4 expression in gastric tumor tissues. *, *P* < 0.05; **, *P* < 0.001. WT, wild-type; MUT, mutant; NC, negative control

Subsequently, we predicted that miR-130a-3p could bind to the position 3121–3127 in the 3ʹUTR of GCNT4 by miRdb analysis (Figure 5a). By transfecting pmiRGLO GCNT4 3ʹ-UTR WT or MUT vectors and miR-130a-3p-NC or miR-130a-3p-mimic into MKN45 and AGS cells, we found significantly reduced (50%) luciferase activities in both cells with GCNT4 3′-UTR WT and miR-130a-3p-mimic, implying that miR-130a-3p targeted GCNT4 (Figure 5b). The RNA-pulldown analysis confirmed the interaction between GCNT4 and miR-130a-3p in both MKN45 and AGS cells (Figure 5c). GCNT4 mRNA and protein expression in gastric tumor tissues were downregulated compared to that in normal tissues (Figure 5d and e). Similarly, the GCNT4 protein level in MKN45 and AGS cells was declined compared with that in normal cells (figure 5f). Finally, we found a negative correlation between miR-130a-3p and GCNT4 expression in tumor tissues (Figure 5g).

### MiR-130a-3p targeting of GCNT4 aggravated the progression of gastric cancer

**Figure 6.**
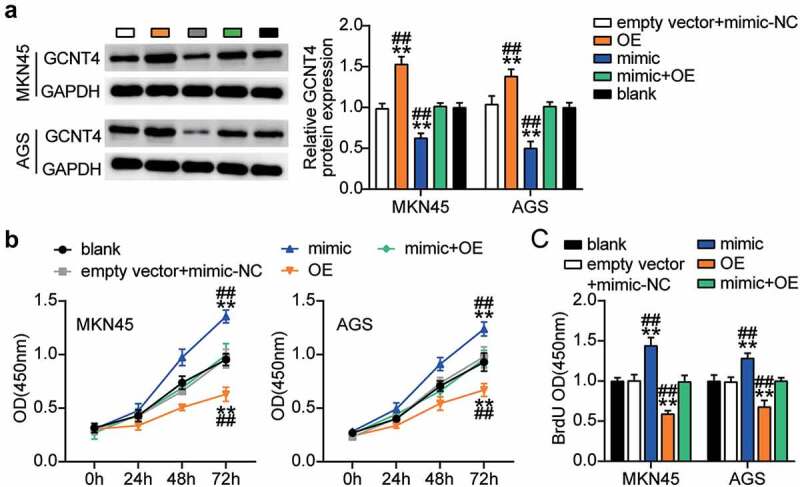
MiR-130a-3p targeting GCNT4 promoted gastric cancer cells growth. (A) Measurement of GCNT4 protein expression in MKN45 and AGS cells transfected with mimic-NC, empty vector, mimic, OE, and OE+ mimic by western blot. (B) Cell viability was detected in MKN45 and AGS cells transfected with mimic-NC, empty vector, mimic, OE, and OE+ mimic by CCK-8 assay. (C) Cell proliferation was detected in MKN45 and AGS cells transfected with mimic-NC, empty vector, mimic, OE, and OE+ mimic by BrdU assay. *, *P* < 0.05; **, *P* < 0.001 compared with blank; ^#^, *P* < 0.05; ^##^, *P* < 0.001 compared with OE+ mimic. NC, negative control; OE, overexpression-GCNT4; mimic, miR-130a-3p mimic; OE+ mimic, overexpression-GCNT4 + miR-130a-3p mimic

**Figure 7.**
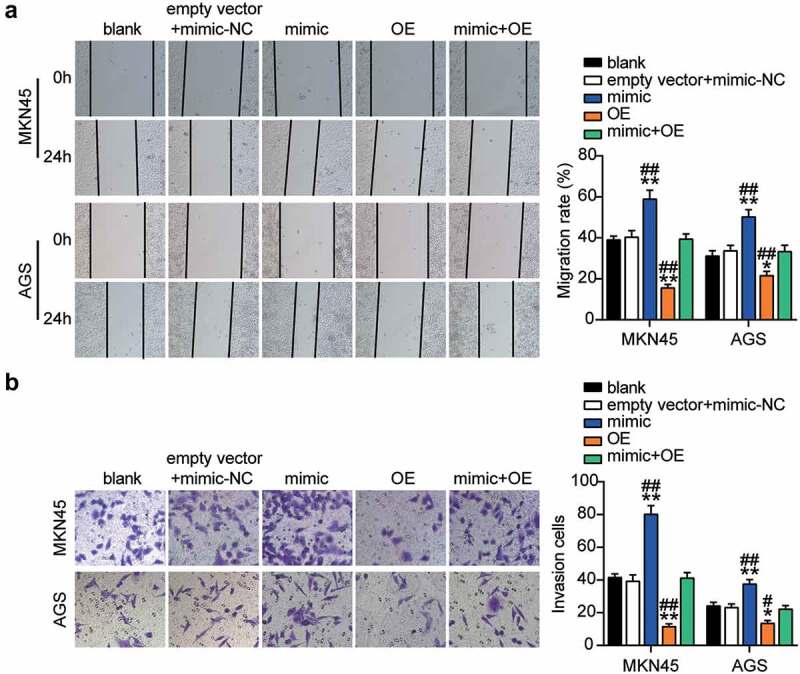
MiR-130a-3p targeting GCNT4 enhanced cell migration and invasion of gastric cancer cells

To elucidate the function of the miR-130a-3p-GCNT4 axis in gastric cancer, we transfected GCNT4 overexpression (OE) and miR-130a-3p-mimic into MKN45 and AGS cells. Mimic groups showed a 50% decrease in GCNT4 protein expression and the OE groups exhibited a 1.5-fold increase in protein expression. OE + mimic transfected groups presented the same level of expression of CGNT4 as in control MKN45 and AGS cells (Figure 6a). The OE groups exhibited significantly higher cell viability than the control cells, whereas this effect was diminished in the OE + mimic transfected groups (Figure 6b). In addition, the OE group indicated 40% downregulation of cell proliferation than in control cells, while the effect was reversed by the OE + mimic treatment (Figure 6c). Furthermore, the OE groups showed 60% and 20% attenuated cell migration compared with control cells in MKN45 and AGS cells, respectively, while the effect was neutralized by the OE + mimic treatment (Figure 7a). Moreover, the OE groups displayed a 60% decrease in cell invasion compared with the control cells, while the outcome was diminished in the OE + mimic groups (Figure 7b). Overall, results showed that targeting of GCNT4 by miR-130a-3p promoted the progression of gastric cancer.

(A) Cell migration level was determined in MKN45 and AGS cells transfected with mimic-NC, empty vector, mimic, OE, and OE+ mimic by wound healing assay. (B) Cell invasion was detected in MKN45 and AGS cells transfected with mimic-NC, empty vector, mimic, OE, and OE+ mimic by transwell assay. *, *P* < 0.05; **, *P* < 0.001 compared with blank; ^#^, *P* < 0.05; ^##^, *P* < 0.001 compared with OE+ mimic. NC, negative control; OE, overexpression-GCNT4; mimic, miR-130a-3p mimic; OE+ mimic, overexpression-GCNT4 + miR-130a-3p mimic.

### Targeting of GCNT4 by miR-130a-3p induced activation of TGF-β1/Smad3 signaling pathway

**Figure 8.**
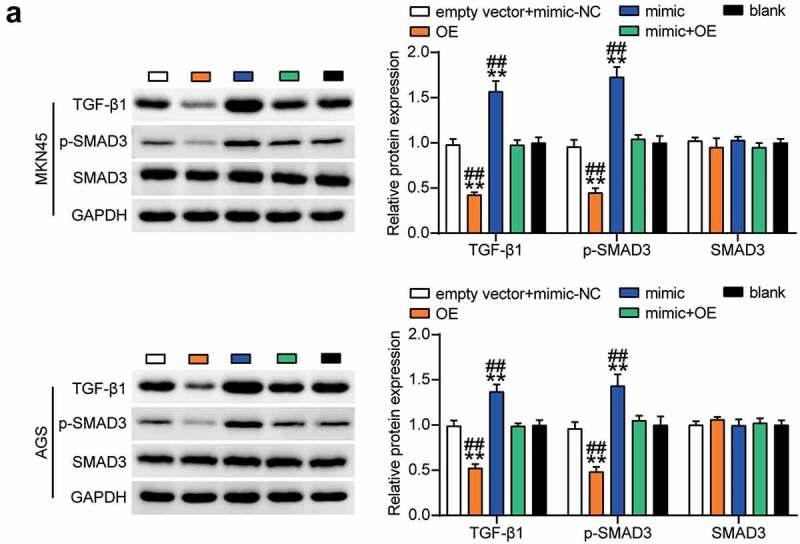
The TGF-β1 and p-smad3 protein expression was detected in MKN45 and AGS cells transfected with mimic-NC, empty vector, mimic, OE, and OE+ mimic by western blot assay. *, *P* < 0.05; **, *P* < 0.001 compared with blank; ^#^, *P* < 0.05; ^##^, *P* < 0.001 compared with OE+ mimic. NC, negative control; OE, overexpression-GCNT4; mimic, miR-130a-3p mimic; OE+ mimic, overexpression-GCNT4 + miR-130a-3p mimic

Next, we investigated the effect of miR-130a-3p mediated regulation of GCNT4 on downstream signaling pathways in MKN45 and AGS cells. Western blot results showed that TGF-β1 and phospho-SMAD3 were downregulated in the OE group compared with those in the control group, and this effect was nullified by the OE + mimic treatment (Figure 8). This indicated that GCNT4 overexpression in gastric cancer cells inhibited the TGF-β1/SMAD3 signaling pathway activated by miR-130a-3p upregulation.

## Discussion

In the present study, elevated miR-130a-3p reinforced gastric cancer cell proliferation, migration, and invasion. Mechanistically, miR-130a-3p was found to facilitate the malignant behavior of gastric cancer by targeting GCNT4. The targeting relationship was supported by the downregulation of GCNT4 expression in gastric cancer tissues and its negative correlation with the miR-130a-3p expression levels. Results revealed the oncogenic role of miR-130a-3p in gastric cancer, suggesting that miR-130a-3p may be a promising target for gastric cancer management.

Multiple studies have shown that miR-130a-3p regulates cellular growth and metastasis in various cancers [8,9,27]. Wang et al. indicated that miR-130a-3p functions as a tumorigenesis-promoting gene in cervical cancer by targeting RUNX family transcription factor 3. Downregulation of miR-130a-3p attenuated cell growth and promoted apoptosis of cervical cancer cells, and overexpression abrogated the effect of miR-130a-3p on tumorigenesis [9]. In contrast, Li et al. have demonstrated that miR-130a-3p acts as a tumor suppressor in renal cell carcinoma, and overexpression of miR-130a-3p inhibited cell growth and migration and enhanced cell apoptosis in renal cancer cells [8]. Kong et al. revealed that miR-130a-3p repressed cell migration of and invasion in human breast cancer stem cell-like cells by inhibiting the member of the RAS oncogene family, RAB5B. Upregulation of miR-130a-3p can prevent tumorigenesis, while downregulation of miR-130a-3p promotes tumorigenesis in BCSCs [27]. Therefore, the role of miR-130a-3p in cancer progression is context-dependent, even in a cellular context. Dai et al. reported that miR-130a-3p was highly expressed in gastric cancer cells and its high expression attenuated the tumor-suppressing role of circGRAMD1B in gastric cancer [10]. Consistently, we also found the overexpression of miR-130a-3p in the GSE93415 data series containing 20 pairs of tissue samples and validated it in 29 pairs of gastric cancer tissues. Furthermore, the tumor-promoting effect of miR-130a-3p was also demonstrated in MKN45 and AGS cells. Nevertheless, Wang et al. have analyzed a GEO database containing 60 primary gastric cancer tissues and eight surrounding noncancerous tissues and reported that miR-130a-3p was poorly expressed in gastric cancer tissues. The discrepancy in miR-130a-3p expression may be associated with the sample size. Therefore, a larger sample size is needed to determine the difference in expression. Wang et al. also examined the effect of miR-130a-3p on gastric cancer cell behavior; miR-130a-3p overexpression in MGC-803 and BGC-823 cells had no effect on gastric cancer proliferation but had an inhibitory effect on gastric cancer cell invasion and migration [11]. These differences might be related to the cell type. In Wang’s investigation, miR-130a-3p inhibitor results in the promotion of SGC-7901 and AGS cell migration and invasion [11]. In the present study, we did not apply the miR-130a-3p inhibitor to test cell behavior. In future work, we will focus on the potential difference.

miRNAs target the 3ʹUTR of mRNA. Using bioinformatics analysis, we found that GCNT4 is a target of miR-130a-3p. GCNT4 is a member of the GCNT family that participates in cancer pathologies, such as colon cancer and pancreatic cancer [13,28]. Chao et al. demonstrated that the upregulation of GCNT2 promoted EMT in colon cancer cells, and its knockdown had the opposite effect [13]. Another study suggested that the downregulation of GCNT3 significantly reduced mucin biosynthesis and pancreatic cancer cell progression [15]. Moreover, the high expression of GCNT3 displayed an antitumor mechanism in both colorectal and pancreatic cancers [28]. Sun et al. found that GCNT4 expression was dramatically decreased in gastric cancer and conferred poor overall survival and disease-free survival to gastric cancer patients. They also overexpressed GCNT4 in gastric cancer cells and found reduced cell proliferation and cell cycle in the cell-line model [16]. In line with previous investigations, we found a low level of GCNT4 in gastric cancer tissues and cells. Upregulation of GCNT4 effectively reduced cell growth in MKN45 and AGS gastric cancer cells. Our RT-qPCR results demonstrated the low expression of GCNT4 in gastric cancer tissues. miR-130a-3p expression was negatively correlated with that of GCNT4, as evidenced from luciferase reporter assays and RT-qPCR. Overexpression of GCNT4 abrogated the promoting effect of miR-130a-3p mimic on the proliferative, migratory, and invasive phenotypes of gastric cancer cells. Accordingly, miR-130a-3p bound 3ʹ UTR of GCNT4 and suppressed the repressing role of GCNT4 on the malignant behavior of gastric cancer cells.

Previous studies have shown that TGF-β1/SMAD3 signaling is involved in the progression of gastric cancer [29]. In addition, miR-130a-3p has been shown to activate the TGF-β1/SMAD3 signaling pathway and induce the phosphorylation of SMAD3 [30]. Similarly, the results of this study showed that miR-130a-3p overexpression upregulated the protein levels of TGF-β1 and p-SMAD3. Additionally, the upregulation of GCNT4 inhibited TGF-β1 expression and SMAD3 phosphorylation. This indicates that miR-130a-3p participates in the regulation of gastric cancer cell behavior by targeting GCNT4 and inducing activation of the TGF-β1/SMAD3 signaling pathway.

miRNAs are sponged by long-chain non-coding RNAs (lncRNAs) containing their responsive elements and negatively regulate mRNA expression at the post-transcriptional level [31]. Previous studies have communicated that the lncRNA-miRNA-mRNA regulatory network plays an important role in gastric cancer. For example, lncRNA HOTAIR, as a competitive endogenous RNA, effectively acts as a sink of miR-331-3p, thus modulating hairy-related 2 inhibition and its expression is positively correlated with gastric cancer progression [32]. HCP5 promotes cell proliferation and induces cell cycle progression through sponge-adsorbed miR-299-3p binding to SMAD family member 5 [22]. Therefore, the upstream regulatory mechanism of the miR-130a-3p-GCNT4 axis needs to be further explored in future studies. More importantly, miRNAs may have different target genes and regulate tumor progression. Our future work will decipher these issues in-depth.

## Conclusions

Overall, this study revealed that miR-130a-3p aggravated gastric cancer cell proliferation, migration, and invasion by reducing GCNT4 expression and activating the TGF-β1/SMAD3 signaling pathway. Therefore, we elucidated the role of the miR-130a-3p/GCNT4/TGF-β1/SMAD3 axis in gastric cancer genesis, which could provide potential therapeutic targets for gastric cancer involving both miR-130a-3p and GCNT4.

## Data Availability

The datasets used and/or analyzed during the current study are available from the corresponding authors on reasonable requests.

## Highlights

- MiR-130a-3p was upregulated, while GCNT4 level was downregulated in gastric cancer
- MiR-130a-3p overexpression facilitated the proliferation, migration, and invasion of gastric cancer cells and activated the TGF-β1/SMAD3 pathway
- Overexpression of GCNT4 prevented cell growth in gastric cancer cells and restrained the activation of the TGF-β1/SMAD3 pathway
- MiR-130a-3p suppressed the genesis of gastric cancer by inhibiting GCNT4 expression and activating the TGF-β1/SMAD3 signaling pathway
